# Gestational Age-Specific Reference Ranges and Internally Validated Z-Score Equations for Fetal Thymus Transverse Diameter at 18 + 0–25 + 6 Weeks of Gestation

**DOI:** 10.3390/diagnostics16182973

**Published:** 2026-09-14

**Authors:** Hasan Beyhekim, Fatma Beyhekim, Zekiye Soykan Sert, Gökçen Örgül, Mete Bertizlioğlu, Aybike Tazegül Pekin

**Affiliations:** 1 Division of Perinatology, Department of Obstetrics and Gynecology, Faculty of Medicine, Selçuk University, 42130 Konya, Türkiye; fatma.beyhekim@selcuk.edu.tr (F.B.); zekiyesoykan@hotmail.com (Z.S.S.); gokcenorgul@gmail.com (G.Ö.); aybiketazegulpekin@gmail.com (A.T.P.); 2 Department of Obstetrics and Gynecology, Faculty of Medicine, Selçuk University, 42130 Konya, Türkiye; mete.bertizlioglu@selcuk.edu.tr

**Keywords:** fetal thymus, reference ranges, centile chart, Z-score, fetal biometry, three-vessel trachea view

## Abstract

**Background/Objectives**: The fetal thymus is increasingly assessed as a marker of fetal wellbeing, yet reliable gestational-age-specific reference data remain limited. We aimed to establish reference ranges, centile charts, and internally validated Z-score equations for the thymus transverse diameter at 18 + 0–25 + 6 weeks of gestation. **Methods**: This retrospective cross-sectional study was conducted at a single tertiary fetal medicine centre between January 2023 and December 2025. Structurally normal singleton pregnancies with a reliable gestational age were eligible. The maximum transverse diameter of the fetal thymus was measured in the three-vessel trachea view. The mean was modelled by polynomial regression and, after heteroscedasticity was confirmed, a gestational-age-specific standard deviation was modelled using the Royston–Wright approach. Z-score equations and smoothed centiles were derived, and performance was assessed by bootstrap, cross-validation, and split-sample validation. **Results**: A total of 2103 pregnancies were included. A cubic mean model was selected, and variance increased significantly with gestation (Breusch–Pagan *p* = 1.34 × 10^−18^). The predicted median increased from 9.7 mm at 18 weeks to 18.3 mm at 25 weeks. Fetal sex was statistically associated with thymus transverse diameter, but the effect was negligible (adjusted mean difference 0.247 mm; *p* = 0.011; Cohen’s d = 0.051; interaction *p* = 0.949), so a single reference was retained. Excluding maternal comorbidities changed the median by at most 0.24 mm. Bootstrap optimism was 0.006 mm and the 10-fold cross-validated root-mean-square error 1.863 mm. **Conclusions**: We provide contemporary gestational-age-specific reference ranges and internally validated Z-score equations for the thymus transverse diameter between 18 + 0 and 25 + 6 weeks, which may support clinical assessment and future research, pending external validation.

## 1. Introduction

The fetal thymus has attracted growing attention as a sonographically accessible marker of fetal wellbeing. A reduction in thymic size has been associated with intra-amniotic inflammation and spontaneous preterm birth, with fetal growth restriction, and with preeclampsia, consistent with the hypothesis that thymic size may reflect an adverse intrauterine environment [1,2,3,4,5]. The thymus is routinely visible in the transverse upper mediastinal planes obtained for cardiac screening [6], and thymic hypoplasia is associated with 22q11.2 deletion syndrome, frequently coexisting with conotruncal malformations [7,8]. Because these planes are already obtained during the standard examination, quantifying the thymus adds little to the protocol, which makes well-founded reference ranges all the more worthwhile. As interest shifts toward the quantitative use of thymic measurements, the clinical value of these observations depends on accurate, gestational-age-specific normative data against which an individual measurement can be judged.

This growing clinical interest has not been matched by consistent normative data. Several reference ranges for the fetal thymus have been reported [9,10,11,12], but they differ considerably in cohort size, measurement definition, gestational coverage, and statistical approach. Many available reference ranges were developed more than a decade ago and were derived from substantially smaller cohorts than those currently achievable through contemporary digital ultrasound archives. As a result, the interpretation of a single thymic measurement may be uncertain, and the same value may be classified differently across reference standards.

Part of this difficulty is methodological. Many published ranges assume a constant standard deviation across gestation, yet the dispersion of thymic measurements widens as pregnancy advances. A notable exception is the cardio-STIC–based study of Pittyanont et al. [12], who modelled the standard deviation of the thymus transverse diameter as an explicit function of gestational age within the same Royston–Wright framework and reported that thymic variability increased with advancing gestation. A single pooled standard deviation therefore tends to misestimate the outer centiles, precisely the P5 and P95 thresholds on which a judgement of an abnormally small or large thymus depends. Modelling the standard deviation as an explicit function of gestational age keeps the centiles correctly calibrated across the whole window of interest and, in turn, enables the derivation of Z-score equations that express thymic size on a continuous, gestation-independent scale. A Z-score offers practical advantages over categorical centile assignment: it permits direct comparison across gestational ages, can be embedded in structured reporting and decision support, and provides the standardized quantity required for research relating thymic size to perinatal outcomes.

Despite these methodological advances, existing mid-second-trimester reference ranges have generally been derived from cohorts considerably smaller than those now available from contemporary digital archives, and formal internal validation of the resulting equations is seldom reported. A reference built from a large contemporary cohort of routine two-dimensional examinations, combining gestational-age-specific variance modelling with bootstrap, cross-validation, and split-sample testing, would therefore complement rather than supersede the existing nomograms. This window coincides with the routine anomaly scan, when the thymus is reliably visualized in the three-vessel trachea view. An up-to-date reference derived from a large single-centre cohort acquired with uniform equipment and a standardized protocol, and accompanied by validated Z-score equations, would provide a practical tool to support both clinical assessment and research. We therefore aimed to establish gestational-age-specific reference ranges, centile charts, and internally validated Z-score equations for the fetal thymus transverse diameter between 18 + 0 and 25 + 6 weeks of gestation.

## 2. Materials and Methods

### 2.1. Study Design and Setting

This was a retrospective cross-sectional study conducted at the Division of Perinatology, Selçuk University Faculty of Medicine, a single tertiary fetal medicine centre, between 1 January 2023 and 12 December 2025. The study was reported in accordance with the STROBE recommendations for cross-sectional studies [13]; the completed checklist is provided as Supplementary Table S1.

### 2.2. Participants and Eligibility

Consecutive singleton pregnancies undergoing routine second-trimester ultrasound during the study period were screened. Eligible pregnancies were singleton, with a live structurally normal fetus, a reliable gestational age between 18 + 0 and 25 + 6 weeks, and an available thymus measurement. Pregnancies were excluded in the presence of multiple gestation, major fetal anomaly, an abnormal prenatal screening or genetic result, intrauterine/fetal growth restriction, a gestational age outside the eligible window, missing thymus measurement, or measurement values that could not be reconciled with the source records. No a priori sample-size calculation was performed; all consecutive eligible records available during the predefined study period were included.

Maternal comorbidities were not used as automatic exclusion criteria. Pregnancies complicated by diabetes, thyroid disease, hypertensive disorders, or conceived by in vitro fertilization were retained in the primary cohort, because their exclusion substantially reduced the sample size, preliminary analyses suggested a minimal effect on thymic size, and their retention may improve external validity. Their influence was instead examined through sensitivity analyses.

### 2.3. Gestational Age and Data Handling

Gestational age was determined from the last menstrual period, with crown–rump length used when a discrepancy existed, and was expressed in days for analysis. During data cleaning, all extreme values were manually reviewed against the original ultrasound records. Fourteen measurements identified as data-entry errors were verified against the original ultrasound records and corrected to the recorded values. Three further values could not be reconciled with the original records and were excluded from further analysis.

### 2.4. Ultrasound Measurement

**Figure 1:**
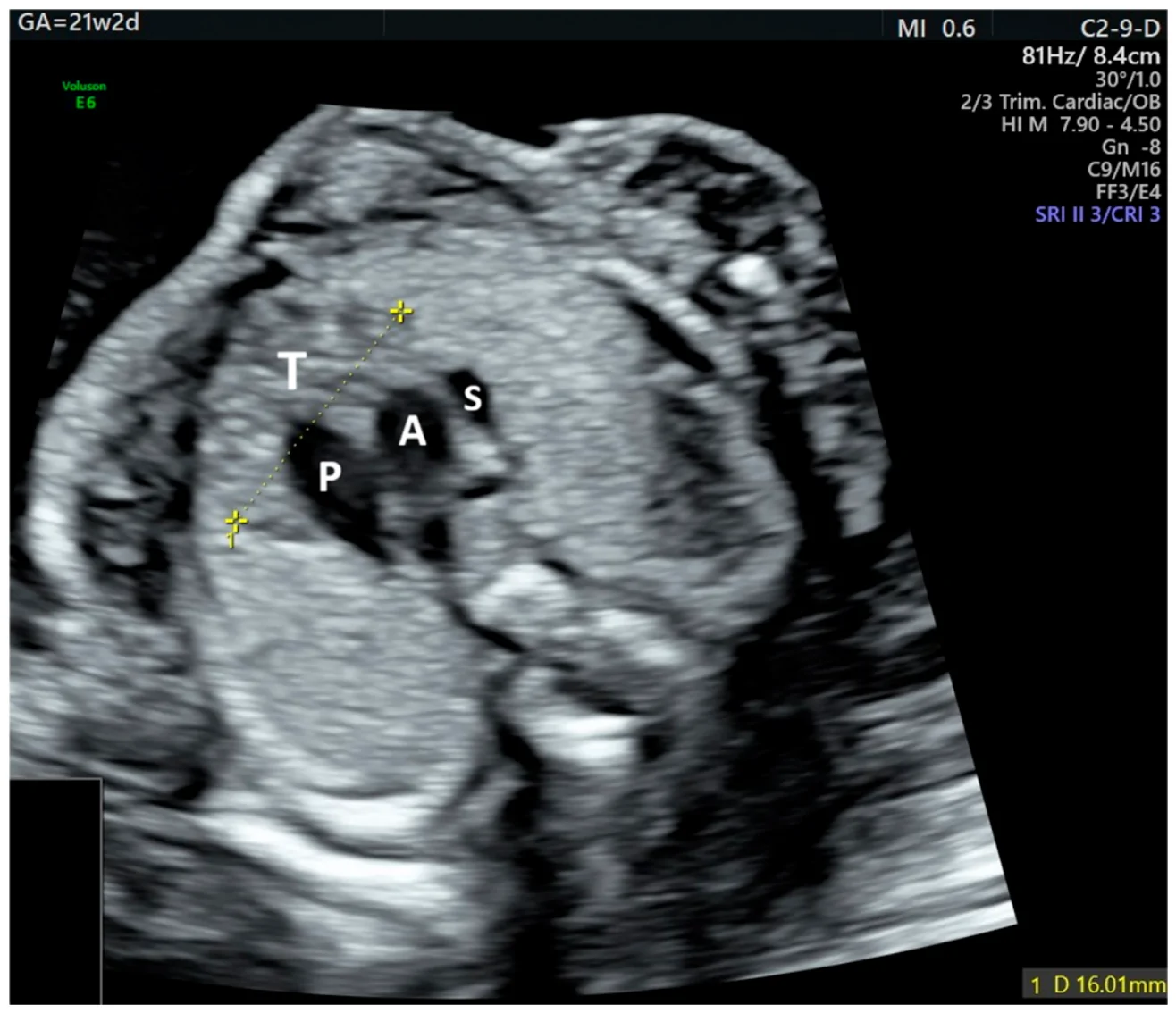
Representative ultrasound image demonstrating measurement of the maximum transverse diameter of the fetal thymus in the three-vessel trachea view. Calipers are positioned at the outer margins of the thymic tissue. T, thymus; A, aorta; P, pulmonary artery; S, superior vena cava.

All examinations were performed using a Voluson E6 BT21 ultrasound system (GE HealthCare, Milwaukee, WI, USA). The fetal thymus was evaluated in the standard three-vessel trachea view. The thymus transverse diameter was defined as the maximum transverse width of the visible thymic tissue. Calipers were placed at the outer margins of the thymic tissue, perpendicular to its longest transverse axis. Measurements were obtained from the thymic parenchyma itself and were not defined by the distance between the internal mammary arteries. Measurements were performed on two-dimensional grayscale (B-mode) images; colour Doppler was not used to delineate the thymic margins. A representative measurement is shown in Figure 1. All measurements were obtained by a single experienced operator using a standardized institutional protocol.

### 2.5. Statistical Analysis

Continuous variables are summarized as mean (standard deviation) or median (interquartile range), and categorical variables as counts and percentages. The relationship between the thymus transverse diameter and gestational age (in days) was modelled by regression; linear, quadratic, and cubic mean models were compared using the Akaike (AIC) and Bayesian (BIC) information criteria, residual diagnostics, and clinical plausibility. Model comparison and heteroscedasticity testing are reported in Section 3.4; the linear mean model is provided as Supplementary Table S2a,b for readers who prefer a more parsimonious specification. A gestational-age-specific standard deviation was modelled using the Royston–Wright approach [14]. Absolute residuals from the fitted mean model were regressed on gestational age, and the resulting fitted values were multiplied by √(π/2) to estimate the gestational-age-specific standard deviation. The Royston–Wright approach was selected because it provides clinically interpretable centiles and Z-scores while remaining consistent with methods used in several established fetal biometric reference studies. Z-scores were derived as the difference between the observed value and the predicted mean divided by the predicted standard deviation, and smoothed centiles (P5, P50, P95) were calculated. The effect of fetal sex was assessed in the 1468 pregnancies with a recorded sex, using an independent-samples *t*-test, the standardized mean difference, analysis of covariance, and a gestational age by sex interaction term; residual variability between male and female fetuses was compared with the Brown–Forsythe test. Model stability was assessed by bootstrap resampling, 10-fold cross-validation, and 70/30 split-sample validation. Bootstrap samples were drawn with replacement at the pregnancy level, and in each resampling iteration and validation split both the selected cubic mean model and the gestational-age-specific variance model were refitted; model selection was not repeated within resamples or folds. One thousand bootstrap resamples were used, optimism was quantified on the root-mean-square error, and percentile-based 95% confidence intervals were calculated for all model coefficients. The 10-fold cross-validation folds and the 70/30 train–test split were generated after random shuffling, and a random seed of 42 was used throughout for reproducibility. No statistical imputation was performed; analyses used the available data for each variable, and pregnancies without a thymus measurement were excluded from the reference-model cohort. Analyses were performed in Python 3.12 (Python Software Foundation, Wilmington, DE, USA) using the open-source NumPy, SciPy, and statsmodels libraries; a two-sided *p* < 0.05 was considered significant. Generative artificial intelligence tools, including Claude Opus 5 (Anthropic, San Francisco, CA, USA), ChatGPT GPT-5.6 Sol (OpenAI, San Francisco, CA, USA), and Gemini 3.1 Pro (Google LLC, Mountain View, CA, USA), were used to assist with verification of data cleaning, development and checking of the statistical code, refitting of the models, generation of the figures, and drafting and English-language editing of the manuscript text, including grammar, clarity, and academic style. The AI tools did not independently make analytical or scientific decisions. All analyses, analytical decisions, interpretations, and reported results were reviewed and verified by the authors.

### 2.6. Sensitivity and Subgroup Analyses

To examine whether retention of maternal comorbidities influenced the reference ranges, five exclusion scenarios were analysed, each applied independently rather than cumulatively: exclusion of all pregnancies complicated by diabetes, by hypertensive disorders, by thyroid disease, by in vitro fertilization (IVF) conception, and by any of these four categories combined. The thyroid-disease scenario was included because maternal thyroid disorders, including subclinical hypothyroidism and Hashimoto thyroiditis, have recently been associated with alterations in fetal thymic measurements, with findings varying according to the thyroid disorder and the thymic metric evaluated [15,16]. The IVF scenario was included because IVF conception has been associated with reduced fetal thymic volume during the second trimester [17]. For each scenario the cubic mean model and the gestational-age-specific variance model were refitted from the remaining pregnancies, and the resulting predicted median, predicted standard deviation, and lower reference limit (P5) were compared with those of the primary cohort. In a separate analysis, pregnancies were assigned to mutually exclusive categories according to these four conditions, and the gestational-age-adjusted Z-scores of each category were compared with those of the comorbidity-free group using Welch’s two-sample *t*-test with Holm step-down correction across the five comparisons; a global comparison across all six categories used Welch’s analysis of variance, with the Kruskal–Wallis test as a non-parametric cross-check.

## 3. Results

### 3.1. Participant Flow

**Figure 2:**
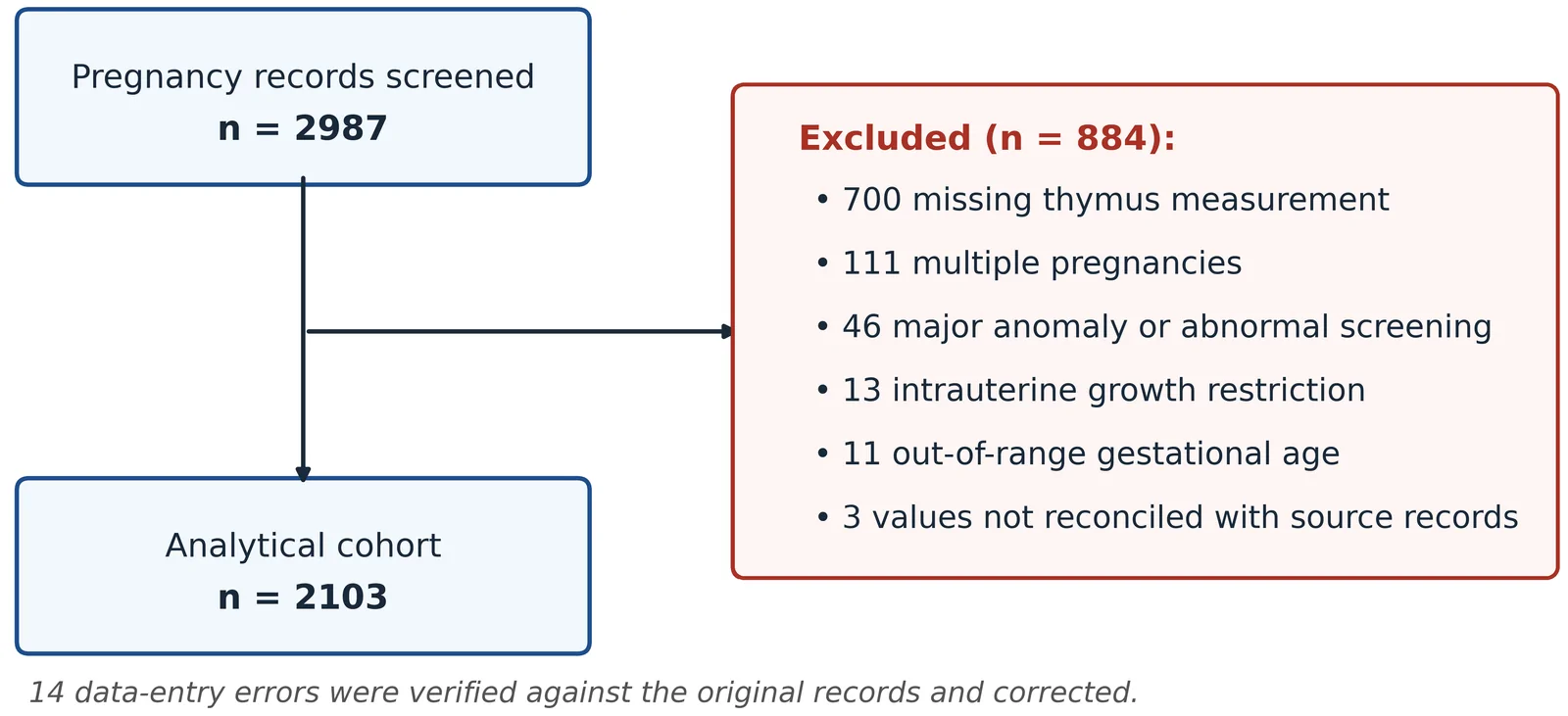
Participant flow diagram (STROBE) showing the screened pregnancy records and the exclusions applied to derive the analytical cohort of 2103 singleton pregnancies.

Of 2987 records, 2103 singleton pregnancies formed the analytical cohort. Exclusions comprised 700 with a missing thymus measurement, 11 with a gestational age outside 18 + 0–25 + 6 weeks, 111 multiple pregnancies, 13 with intrauterine growth restriction, and 46 with a major anomaly or abnormal screening; a further 3 values that could not be reconciled with the original records were removed, while 14 data-entry errors were verified against the original records and corrected rather than excluded (Figure 2).

### 3.2. Cohort Characteristics

Maternal and pregnancy characteristics are summarized in Table 1. The mean maternal age was 29.2 ± 4.8 years, and just over half of the women were nulliparous. Examinations were performed at a mean gestational age of 21.5 ± 1.3 weeks. The most frequent maternal condition was thyroid disease (245; 11.7%), followed by diabetes (56; 2.7%), hypertensive disorders (37; 1.8%), and IVF conception (55; 2.6%). Fetal sex was recorded in 1468 fetuses, of which approximately 52% were male.

### 3.3. Observed Measurements

The observed thymus transverse diameter increased monotonically with gestation, from a mean of 9.78 mm at 18 weeks to 18.24 mm at 25 weeks (observed values, Table 2). The weekly standard deviation ranged from 1.44 mm to 2.88 mm across gestation.

### 3.4. Model Selection and Equations

Linear, quadratic, and cubic mean models produced closely comparable fit (AIC 8586.7, 8584.8, and 8582.3; adjusted R^2^ = 0.386–0.387; RMSE 1.86 mm). The cubic model provided the lowest AIC and differed significantly from the linear specification on likelihood-ratio testing (*p* = 0.015), although the Bayesian Information Criterion favoured the linear model (Supplementary Table S2a,b); the cubic model was retained because it also offered a biologically plausible representation of non-linear thymic growth, and yielded centiles within 0.66 mm of the linear model. The Breusch–Pagan test confirmed heteroscedasticity (*p* = 1.34 × 10^−18^), so a gestational-age-specific standard deviation was modelled. The final mean, standard deviation, and Z-score equations are given in Table 3.

### 3.5. Smoothed Reference Ranges

**Figure 3:**
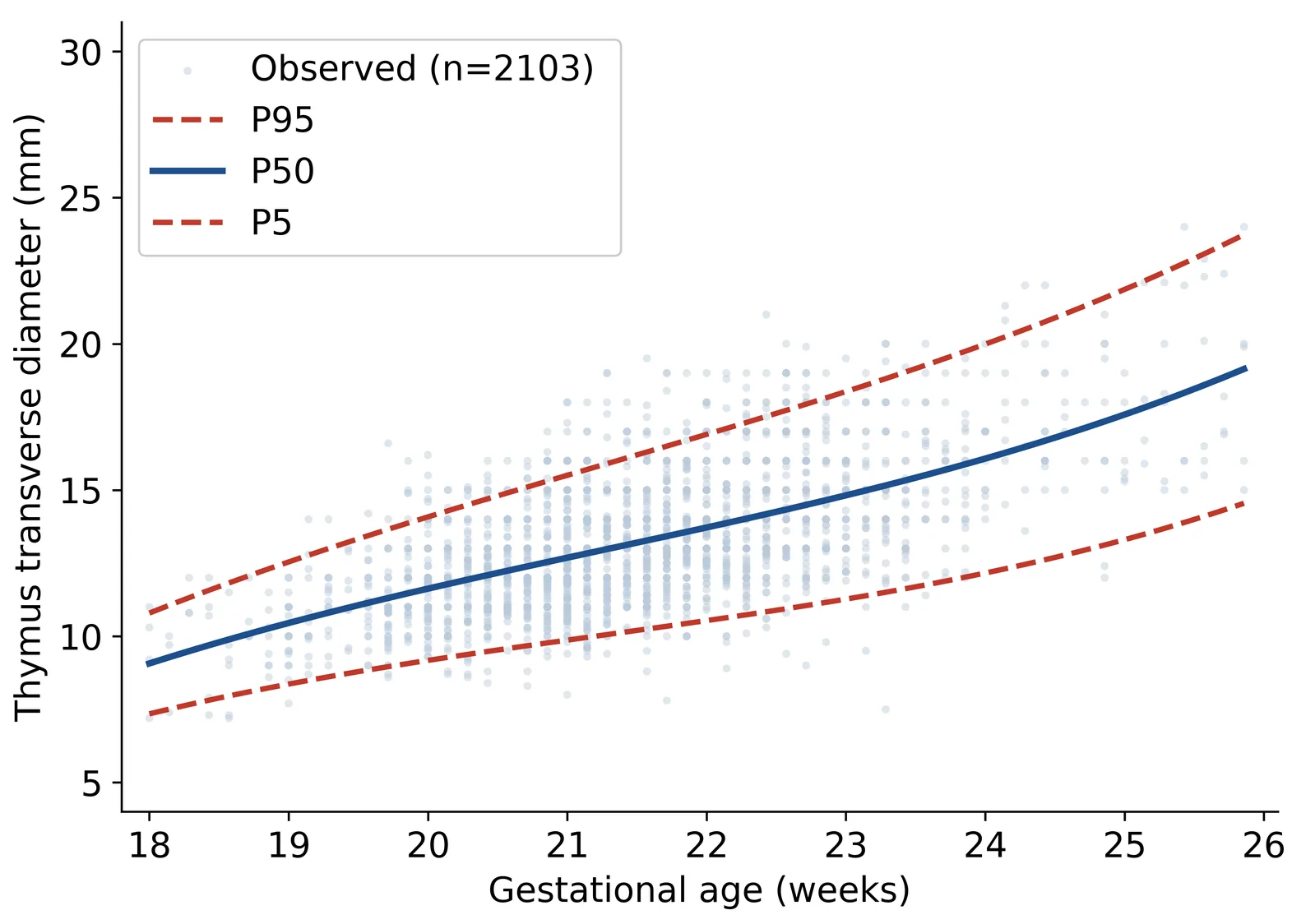
Observed versus smoothed (predicted) 5th, 50th, and 95th centiles of the fetal thymus transverse diameter (mm) across gestational age (weeks).

Model-derived (predicted) centiles are presented in Table 4 and Figure 3. The predicted median increased from 9.69 mm at 18 weeks to 18.33 mm at 25 weeks, with the reference interval widening accordingly. Observed (Table 2) and predicted (Table 4) centiles agreed closely. Figure 3 shows the individual observations together with the fitted centile curves.

### 3.6. Z-Score Diagnostics

**Figure 4:**
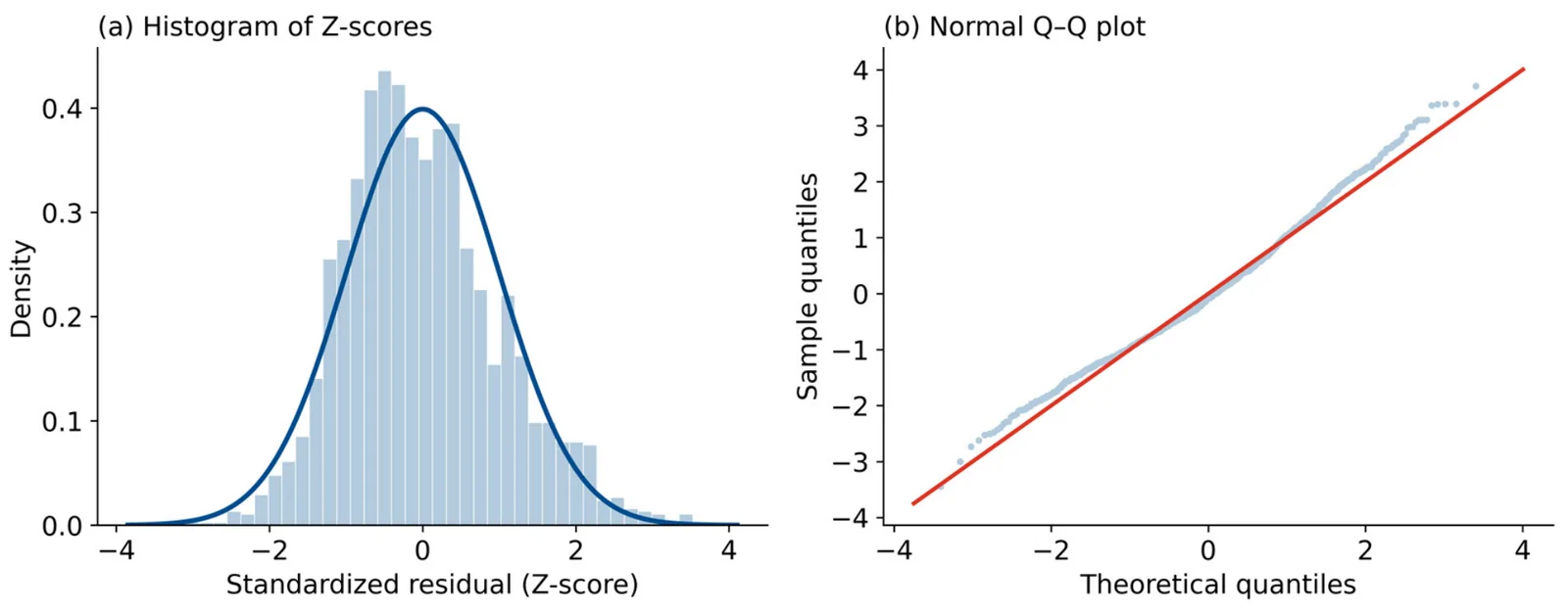
Residual and Z-score diagnostics: (**a**) histogram of standardized residuals with the standard normal density overlaid; (**b**) normal quantile–quantile plot of the derived Z-scores.

The standardized residuals showed mild positive skewness (0.44); the empirical 5th and 95th centiles of the Z-scores were −1.45 and +1.84 against the expected values of −1.645 and +1.645, indicating broadly adequate, though not perfect, normality. The standard deviation of the derived Z-scores was 1.005, indicating that the variance model captured the dispersion of the data closely (Figure 4).

### 3.7. Coefficient Confidence Intervals

Percentile-based bootstrap 95% confidence intervals for all mean- and variance-model coefficients are given in Supplementary Table S3a,b. The slope coefficient of the variance model excluded zero (0.025294, 95% CI 0.019581 to 0.031019), supporting gestational-age-specific variance modelling; the intercept also excluded zero (−2.351178, 95% CI −3.183274 to −1.503333). Bootstrap confidence intervals for the individual polynomial mean-model coefficients were wide and crossed zero, reflecting collinearity among the polynomial terms; the fitted central trajectory, however, remained stable across resamples (Supplementary Table S3c), with a 95% confidence interval width of 0.17–0.37 mm between 21 and 23 weeks, widening to 0.84 mm at 18 weeks and 1.53 mm at 25 weeks where weekly samples were smallest.

### 3.8. Fetal Sex

A statistically significant association between fetal sex and thymus transverse diameter was observed; however, the absolute difference was small and the standardized effect was negligible (adjusted mean difference, male minus female, 0.247 mm; *p* = 0.011; Cohen’s d = 0.051). The gestational age by sex interaction was not significant (*p* = 0.949), indicating no evidence of sex-specific growth trajectories across the studied gestational window. Residual variability was comparable between male and female fetuses (residual SD, 1.85 mm in males vs. 1.84 mm in females; Brown–Forsythe *p* = 0.658). Given the negligible effect size, the comparable residual variability, and the absence of an interaction with gestational age, a single reference model was retained for both sexes.

### 3.9. Sensitivity Analyses

Independent exclusion of pregnancies with thyroid disease, diabetes, hypertensive disorders, IVF conception, or all comorbidities combined changed the predicted median by no more than 0.24 mm across the whole gestational window; the largest shift followed exclusion of all comorbidities combined (*n* = 370 excluded), and exclusion of any single condition altered the median by ≤0.16 mm. No single-condition exclusion shifted the lower reference boundary (P5, calculated as μ(t) − 1.645·σ(t)) by more than 0.18 mm, and combined exclusion of all four categories shifted it by at most 0.27 mm; in every scenario the maximum shift occurred at 25 weeks (Supplementary Table S4). Mean Z-scores did not differ significantly between the comorbidity-free reference group and any maternal-condition subgroup (all *p* > 0.05, Welch’s *t*-test), and no subgroup showed a statistically significant trend with gestational age, although the hypertensive-disorder subgroup showed the largest negative slope (−0.308 Z per week; *p* = 0.061), which should be interpreted cautiously given the small subgroup size (Supplementary Table S5).

### 3.10. Internal Validation

Internal validation indicated stable performance with minimal optimism: the apparent root-mean-square error was 1.858 mm (apparent R^2^ = 0.39), the 10-fold cross-validated error was 1.863 mm, and the 70/30 split-sample test error was 1.862 mm. Bootstrap resampling indicated an optimism of 0.006 mm, giving an optimism-corrected error of 1.864 mm. Held-out Z-scores retained a standard deviation close to unity under both cross-validation (1.010) and split-sample validation (1.011), indicating that the variance model generalized as well as the mean model.

## 4. Discussion

In this large single-centre study of 2103 structurally normal singleton pregnancies, we established gestational-age-specific reference ranges, centile charts, and internally validated Z-score equations for the fetal thymus transverse diameter between 18 + 0 and 25 + 6 weeks of gestation. The thymus enlarged predictably across this window, with the predicted median increasing from approximately 9.7 mm at 18 weeks to approximately 18.3 mm at 25 weeks, and a cubic function provided the most appropriate description of the mean. The dispersion of measurements increased significantly with advancing gestation, so a constant standard deviation was inappropriate and the standard deviation was instead modelled as an explicit function of gestational age. Fetal sex was statistically associated with thymus transverse diameter, but the difference was too small to justify separate references, and the reference ranges were robust to the inclusion of common maternal comorbidities. Internal validation indicated stable model performance and adequate internal validity.

Our results are consistent with the established pattern of progressive thymic growth during the second trimester. Published transverse-diameter nomograms differ considerably. Our reference ranges lay toward the lower end of the published range, below those reported by Cho et al. [9] and by Tangshewinsirikul and Panburana [10], and may partly reflect differences in measurement definition. Cho et al. derived their reference range from a smaller cohort spanning a wider gestational range, whereas Tangshewinsirikul and Panburana reported larger absolute values across 17 to 38 weeks. The systematically lower values in our cohort may partly reflect our strict definition of the measurement as the thymic parenchyma alone, excluding the surrounding perithymic space included by wider landmark-based methods. This discrepancy most plausibly reflects differences in measurement landmarks, imaging technique, study population, and statistical methodology rather than error in any single dataset. In particular, measurements based on the inter–mammary-artery “thy-box” width [6] may not be directly interchangeable with measurements defined as the maximum transverse diameter of thymic tissue. This heterogeneity reinforces the need for internally consistent, technique-specific contemporary reference ranges accompanied by Z-score equations. We do not regard our reference ranges as superseding earlier nomograms.

Our reference values were also lower than those reported by Pittyanont et al. [12], whose 50th-centile values exceeded ours by approximately 1.5 mm at 18 weeks and 4.6 mm at 25 weeks. Their measurements were obtained in the three-vessel view from cardio-STIC volume datasets that could be manipulated offline to identify the plane of maximal thymic diameter, whereas our measurements were obtained using two-dimensional imaging in the three-vessel trachea view. Zych-Krekora et al. [11] similarly developed nomograms from the three-vessel transverse view in 410 fetuses between 14 and 40 weeks and evaluated transverse diameter together with circumference and surface area. Their approach used linear regression for thymic diameter, whereas Pittyanont et al. and the present study explicitly modelled gestational-age-related variability to derive standardized reference values. Differences in imaging plane, acquisition technique, study population, gestational-age coverage, and statistical modelling may therefore contribute to the differences observed among published reference ranges.

We report the maximum transverse diameter because it is the parameter reported in common across the comparator studies discussed above and because Pittyanont et al. recommended it as the simplest and most reproducible thymic measurement [12].

Fetal thymic biometry has been evaluated in the literature using both the three-vessel view (3VV) and the three-vessel trachea view (3VT), indicating that both upper mediastinal planes can be used for sonographic assessment of the fetal thymus [6,11,12,18,19]. Myers et al. evaluated thymic measurements in both the 3VV and 3VT in the same fetuses and reported that the thymus transverse diameter measured in the 3VT correlated significantly with MRI-derived thymic volume (r = 0.544, *p* = 0.002) [19]. In the present study, the 3VT was selected because it forms part of our institutional routine second-trimester fetal cardiac screening protocol and permits assessment of the thymus anterior to the great vessels. This choice is also clinically relevant because assessment of the fetal thymus is particularly pertinent in the setting of conotruncal anomalies and suspected 22q11.2 deletion. Accordingly, incorporating transverse thymic measurement into the routinely acquired 3VT image may provide a practical means of assessing whether thymic size is appropriate for gestational age without requiring an additional imaging plane.

Expressing thymic size as a Z-score places the measurement on a continuous, gestation-independent scale and offers practical advantages over categorical centile assignment. For example, a thymus measuring 10.0 mm at 21 weeks corresponds to Z = (10.0 − 13.13)/1.81 = −1.73, that is, just below the 5th centile. A Z-score allows the same fetus to be tracked across gestational ages on a common, gestation-independent scale; however, because this is a cross-sectional study, it describes population dispersion at each gestational age rather than each fetus’s own longitudinal growth trajectory, and does not by itself constitute a validated longitudinal reference for serial thymus surveillance. It can be incorporated into structured reporting, and provides a standardized quantity that facilitates research and comparison across centres, for example in studies relating thymic size to intra-amniotic inflammation, fetal growth restriction, maternal diabetes and thyroid disease, or conotruncal and microdeletion-associated cardiac disease. Fetal thymic measurements have also been evaluated in congenital heart disease, where a reduced thymic–thoracic ratio has been reported in conotruncal defects [20] and has been associated with adverse perinatal outcomes [21]. More recent second-trimester data also suggest that fetal thymic measurements may be associated with subsequent impaired fetal growth; in a cohort assessed at 19 + 0–21 + 6 weeks, fetuses later born small for gestational age had higher thymic–thoracic ratios than controls [22]. Other second-trimester studies have reported null or opposite associations [18], underscoring the need for standardized measurement and robust reference data. Because the three-vessel trachea plane is already acquired during the routine anomaly scan performed in this gestational window, the additional burden of quantifying the thymus is small. These reference ranges describe expected size at a given gestational age and are intended to support, rather than replace, comprehensive fetal assessment; an individual value outside the expected range should prompt correlation with the wider clinical and sonographic picture rather than serving as a diagnostic threshold in isolation.

The principal strengths of this study are its large and contemporary cohort, drawn from a single centre with uniform equipment and a standardized measurement protocol, which limits between-operator and between-device heterogeneity. The sample is considerably larger than those underlying several earlier reference ranges, and the exclusion of multiple pregnancies, major anomalies, and growth-restricted fetuses yields a structurally normal, non-growth-restricted reference cohort. Gestational age was analysed in days rather than completed weeks, preserving resolution across the study window. The explicit modelling of gestational-age-specific variance, rather than the assumption of a constant standard deviation, yields centiles that remain calibrated throughout gestation, and the sensitivity analyses indicate that the reference ranges are only minimally affected by common maternal comorbidities. Although fetal sex was statistically associated with thymus transverse diameter, the absolute difference was small and the standardized effect was negligible; there was no evidence of a sex-specific gestational trajectory, and residual variability was comparable between male and female fetuses, so separate sex-specific reference equations were not considered clinically or statistically justified. Finally, the use of three complementary internal validation approaches supports the stability and internal validity of the derived equations. The negligible bootstrap optimism and nearly identical apparent and cross-validated errors suggest minimal overfitting and support the robustness of the derived equations.

Several limitations should be acknowledged. This retrospective archive-based study did not include a formal reproducibility assessment. All measurements were obtained by a single experienced operator using a standardized three-vessel trachea protocol; nevertheless, the absence of study-specific intra- and inter-observer intraclass correlation coefficient data should be considered a limitation. The single-centre design may limit generalizability to other populations and equipment. Because the cohort was assembled from a clinical ultrasound archive, the analysis was restricted to pregnancies in which a thymus measurement had been recorded, which could introduce a degree of selection: sonographers may have been more likely to document a thymus measurement when the view was readily obtained or the gland appeared unremarkable, and less likely when imaging was technically difficult or the appearance was atypical. This is an important limitation, and the reported reference ranges should be interpreted with this potential selection bias in mind, particularly at the outer centiles. Maternal comorbidities were deliberately retained to improve external validity; although sensitivity analyses showed their effect to be minimal, some readers may prefer reference ranges derived from an entirely uncomplicated population. Because maternal conditions that have been associated with altered fetal thymic measurements were retained rather than excluded, the reference ranges reflect the clinical case mix of the source population. However, the per-condition sensitivity analyses showed only small and directionally variable shifts in the fitted reference values, suggesting limited influence on the derived ranges (Supplementary Tables S4,5). Reduced fetal thymus size has been reported in pregnancies complicated by maternal diabetes, although the findings appear to depend on the measurement used. Reductions have been described using the anteroposterior thymic diameter [23,24] and using the anteroposterior-derived thymic–thoracic ratio and thymic circumference [25], whereas findings based on the maximum transverse diameter are inconsistent. Sinaci and Sahin reported smaller fetal thymic measurements, including the transverse diameter, in pregnancies complicated by gestational or pregestational diabetes [26], whereas Gök and Özden found a significantly lower thymic–thoracic ratio in gestational diabetes but no significant difference in transverse diameter [27]. Mansouri et al. also discussed differences in thymic measurement methodology as a potential explanation for discrepancies among studies [25]. This heterogeneity across measurement approaches and reported associations underscores the need for gestational-age-specific reference values derived from a single, explicitly defined measurement. Our subgroup findings for transverse diameter should be interpreted in this context and do not exclude an effect on other thymic parameters. Maternal body mass index, smoking status, and ethnicity were not recorded in the archive and could not be examined. However, recent studies have reported no significant association between maternal body mass index and fetal thymic measurements [28,29], suggesting that the absence of body mass index data was unlikely to have materially influenced the derived reference ranges. Fetal sex was unavailable in approximately one-third of the cohort, so the sex analyses were restricted to the 1468 pregnancies with a recorded sex. Although a cubic mean model was selected, simpler polynomial models fitted comparably, and the reference ranges should be regarded as a pragmatic description of central tendency rather than evidence of a specific functional form. Importantly, the resulting centiles differed by no more than 0.66 mm between the linear and cubic models. The Z-score distribution showed mild positive skewness, so a threshold of Z < −1.645 identifies slightly fewer than 5% of fetuses at the lower extreme; the empirical centiles in Table 2 are therefore presented alongside the modelled values. Gestational age was determined primarily from the last menstrual period, with crown–rump length used where a discrepancy existed. The equations apply specifically to 18 + 0–25 + 6 weeks and should not be extrapolated beyond this window, and the smallest weekly sample sizes occurred at the boundaries of the study range, so centile estimates at 18 and 25 weeks should be interpreted with greater caution than those in the central gestational weeks. Internal validation was conditional on the selected functional forms, because model selection was not repeated within resamples or folds. As a cross-sectional study, our data describe thymic size at a given gestational age rather than individual longitudinal growth, and external validation in independent populations represents an important direction for future work.

## 5. Conclusions

This study provides contemporary, gestational-age-specific reference ranges, centile charts, and internally validated Z-score equations for the fetal thymus transverse diameter between 18 + 0 and 25 + 6 weeks of gestation, derived from a large cohort of singleton pregnancies. By combining a contemporary dataset with gestational-age-specific variance modelling, extensive internal validation, and clinically applicable Z-score equations, these reference ranges may offer a sound, contemporary normative basis for both clinical assessment and future research into fetal thymic development, pending external validation in independent populations.

## Figures and Tables

**Table 1:** Cohort characteristics (*n* = 2103).

Characteristic	Value
Maternal age, years	29.2 ± 4.8 (median 29, IQR 26–32; range 18–47)
Gravidity, median (IQR)	2 (1–2)
Parity, median (IQR)	0 (0–1)
Nulliparous, *n* (%) ^a^	1121 (54.8)
Gestational age at examination, weeks	21.5 ± 1.3
Male fetus, *n* (%) of recorded sex ^b^	767/1468 (52.2)
Thyroid disease, *n* (%)	245 (11.7)
Diabetes/GDM, *n* (%)	56 (2.7)
Hypertensive disorders, *n* (%)	37 (1.8)
IVF conception, *n* (%)	55 (2.6)
Aspirin/heparin, *n* (%)	215 (10.2)

^a^ Parity was recorded in 2047 pregnancies; the nulliparous percentage refers to this denominator. ^b^ Fetal sex was not recorded in 635 (30.2%); percentages refer to the 1468 with recorded sex. Abbreviations: GDM, gestational diabetes mellitus; IQR, interquartile range; IVF, in vitro fertilization; SD, standard deviation.

**Table 2:** Observed weekly reference values for fetal thymus transverse diameter (mm) (*n* = 2103).

GA (wk)	*n*	Mean (mm)	SD (mm)	P5	P50	P95
18	34	9.78	1.44	7.26	10.00	12.00
19	150	11.15	1.53	9.00	11.00	14.00
20	489	12.06	1.57	9.84	12.00	15.00
21	699	13.14	1.84	10.40	13.00	16.02
22	454	14.21	2.11	11.30	14.00	18.00
23	186	14.92	2.15	12.00	15.00	19.00
24	55	17.17	2.33	13.88	17.00	21.09
25	36	18.24	2.88	15.00	17.50	23.17

Empirical (observed) percentiles; descriptive only. Abbreviations: GA, gestational age; P5, 5th percentile; P50, 50th percentile; P95, 95th percentile; SD, standard deviation.

**Table 3:** Final equations and model comparison for fetal thymus transverse diameter.

Component	Equation/Value
Mean model	μ(t) = −155.712390 + 3.104289·t − 0.01985762·t^2^ + 0.0000444422·t^3^
SD model	σ(t) = (−2.3512 + 0.025294·t) × √(π/2)
Z-score	Z = (observed − μ(t))/σ(t) [t = GA in days]
Model comparison	AIC: linear 8586.7, quadratic 8584.8, cubic 8582.3; BIC: linear 8598.0, quadratic 8601.8, cubic 8604.9; adj-R^2^ 0.386–0.387; RMSE 1.86 mm; cubic vs. linear likelihood-ratio *p* = 0.015

Abbreviations: AIC, Akaike Information Criterion; BIC, Bayesian Information Criterion; GA, gestational age; RMSE, root-mean-square error; SD, standard deviation. t denotes gestational age in days; adj-R^2^, adjusted coefficient of determination; μ(t), modelled mean function; σ(t), modelled standard-deviation function.

**Table 4:** Smoothed, model-derived (predicted) reference ranges for fetal thymus transverse diameter (mm).

GA (wk)	t (Days)	Pred. Mean	Pred. SD	P5	P50	P95
18	129	9.69	1.14	7.81	9.69	11.57
19	136	10.98	1.36	8.73	10.98	13.22
20	143	12.09	1.59	9.48	12.09	14.70
21	150	13.13	1.81	10.15	13.13	16.10
22	157	14.18	2.03	10.84	14.18	17.52
23	164	15.33	2.25	11.63	15.33	19.04
24	171	16.68	2.47	12.61	16.68	20.75
25	178	18.33	2.70	13.89	18.33	22.76

Centiles calculated as μ(t) ± 1.645·σ(t); t = gestational age in days at the midpoint of each week. Abbreviations: GA, gestational age; P5, 5th percentile; P50, 50th percentile; P95, 95th percentile; Pred., predicted; SD, standard deviation.

## Data Availability

The data presented in this study are available on reasonable request from the corresponding author due to patient privacy and confidentiality restrictions and subject to approval by the relevant institutional ethics committee.

## Supplementary Materials

The following supporting information can be downloaded at: https://www.mdpi.com/article/10.3390/diagnostics16182973/s1. Table S1: STROBE checklist for cross-sectional studies; Table S2: Linear mean-model equation and corresponding smoothed centiles for the fetal thymus transverse diameter (S2a,b); Table S3: Bootstrap 95% confidence intervals for the mean-model coefficients (S3a), the variance-model coefficients (S3b), and the fitted median curve (S3c). Table S4: Predicted median, predicted standard deviation, and lower reference limit (P5) for the primary cohort and for each independent exclusion scenario, with the change in P5 relative to the primary cohort (diabetes, hypertensive disorders, thyroid disease, IVF conception, and all four categories combined); Table S5: Gestational-age-adjusted fetal thymus Z-scores by mutually exclusive maternal comorbidity category, including mean differences versus the comorbidity-free reference group, 95% confidence intervals, multiplicity-adjusted comparisons, and gestational-age trends.
