# Supplementary material for: Gestational Age-Specific Reference Ranges and Internally Validated Z-Score Equations for Fetal Thymus Transverse Diameter at 18 + 0–25 + 6 Weeks of Gestation
Source: Diagnostics (Basel). 2026 Sep 14;16(18):2973. doi: 10.3390/diagnostics16182973 (PMC13605929; doi:10.3390/diagnostics16182973)
Supplement: Supplementary file 1 [file diagnostics-16-02973-s001.zip › 04_Supplementary_S1_STROBE_Checklist.pdf]

Table S1. STROBE Statement — Checklist for cross-sectional studies

Manuscript: Gestational Age–Specific Reference Ranges and Internally Validated Z-Score Equations for Fetal Thymus Transverse Diameter at 18+0–25+6 Weeks of Gestation

Section / Topic	Item	Recommendation	Reported in (section)
**Title and abstract**	1	(a) Study design in title/abstract; (b) balanced summary in abstract	Abstract (Background/Objectives, Methods, Results, Conclusions) — p. 1
**Introduction**			
**Background/rationale**	2	Scientific background and rationale	Introduction, para. 1–2 — p. 2
**Objectives**	3	Specific objectives	Introduction, final para. — p. 2
**Methods**			
**Study design**	4	Key elements of design early in paper	Methods 2.1 — Study Design and Setting — p. 3
**Setting**	5	Setting, locations, relevant dates	Methods 2.1 — Study Design and Setting — p. 3
**Participants**	6	Eligibility criteria, sources, selection	Methods 2.2 — Participants and Eligibility — p. 3; Figure 2 (flow) — p. 6
**Variables**	7	Outcomes, exposures, predictors, confounders	Methods 2.3–2.4; Results 3.2, Table 1 — pp. 3–4, 6
**Data sources/measurement**	8	Sources and measurement methods; comparability	Methods 2.4 — Ultrasound Measurement (3VT view, GE Voluson E6) — p. 4; Figure 1 — p. 4
**Bias**	9	Efforts to address potential bias	Methods 2.2 (eligibility and exclusions), 2.3 (source-record verification of extreme values), 2.6 (sensitivity analyses) — pp. 3–5
**Study size**	10	How study size was arrived at	Methods 2.2 — no a priori sample-size calculation; all consecutive eligible records included — p. 3; Figure 2 (n = 2,103 from 2,987) — p. 6
**Quantitative variables**	11	Handling of quantitative variables/groupings	Methods 2.5 — Statistical Analysis (gestational age in days; centiles and Z-scores) —

			pp. 4–5
**Statistical methods**	12	(a) Methods incl. confounding; (b) subgroups; (c) missing data; (d) sampling; (e) sensitivity	Methods 2.5 — model selection, Royston–Wright SD, Z-scores, Brown–Forsythe test, bootstrap/CV/split-sample validation; no imputation, available-data analysis, pregnancies without a thymus measurement excluded — pp. 4–5; Methods 2.6 — sensitivity analyses — p. 5
**Results**			
**Participants**	13	(a) Numbers at each stage; (b) reasons for non-participation; (c) flow diagram	Results 3.1 — Participant Flow — p. 6; Figure 2 — p. 6
**Descriptive data**	14	Characteristics of participants; missing data	Results 3.2 — Cohort Characteristics — p. 6; Table 1 (missing parity and fetal sex in footnotes) — pp. 6–7
**Outcome data**	15	Report numbers of outcome events/summary measures	Results 3.3 — Table 2 (observed weekly values) — p. 7; Results 3.5 — Table 4 (smoothed model-derived centiles) — p. 8
**Main results**	16	Estimates and precision; category boundaries	Results 3.4–3.5 — Table 3 (equations) — pp. 7–8; Table 4 (centiles) — p. 8; Figure 3 — p. 9; Results 3.7 — Table S3 — p. 9
**Other analyses**	17	Other analyses (subgroups, interactions, sensitivity)	Results 3.6–3.10 — Z-score diagnostics, fetal sex, sensitivity analyses, internal validation — pp. 9–10; Figure 4 — p. 9
**Discussion**			
**Key results**	18	Summarise key results with reference to objectives	Discussion, opening paragraph
**Limitations**	19	Limitations, sources of bias/imprecision	Discussion — Limitations paragraph
**Interpretation**	20	Cautious overall interpretation	Discussion — interpretation & comparison paragraphs

**Generalisability**	21	External validity	Discussion — single-centre, technique-specific caveats
**Other information**			
**Funding**	22	Source of funding and role of funders	Declarations — Funding

Locations refer to numbered manuscript sections and to page numbers in the revised manuscript.
