# Supplementary material for: Gestational Age-Specific Reference Ranges and Internally Validated Z-Score Equations for Fetal Thymus Transverse Diameter at 18 + 0–25 + 6 Weeks of Gestation
Source: Diagnostics (Basel). 2026 Sep 14;16(18):2973. doi: 10.3390/diagnostics16182973 (PMC13605929; doi:10.3390/diagnostics16182973)
Supplement: Supplementary file 1 [file diagnostics-16-02973-s001.zip › 05_Supplementary_S2-S3.pdf]

Supplementary Materials

Gestational Age–Specific Reference Ranges and Internally Validated Z-Score Equations for Fetal Thymus Transverse Diameter at 18+0–25+6 Weeks of Gestation

Table S1. STROBE checklist

The completed STROBE checklist for cross-sectional studies is submitted as a separate file in accordance with the reporting guidance cited in the Materials and Methods section.

Table S2. Linear mean-model specification

The cubic mean model was retained in the main analysis because it provided the lowest Akaike Information Criterion and differed significantly from the linear specification on likelihood-ratio testing ($p = 0.015$). The Bayesian Information Criterion, however, favoured the more parsimonious linear model. The linear specification is provided here as a transparency and sensitivity comparison; the cubic model remains the primary model for reporting. Across the study window the two models produced median values differing by no more than 0.66 mm.

Table S2a. Linear mean and standard-deviation equations for fetal thymus transverse diameter.

Component	Equation
Mean model	$\mu(t) = -11.038121 + 0.161260 \cdot t$
SD model	$\sigma(t) = (-2.4399 + 0.025897 \cdot t) \times \sqrt{(\pi/2)}$
Z-score	$Z = (\text{observed} - \mu(t)) / \sigma(t)$

t denotes gestational age in days.

Table S2b. Smoothed centiles derived from the linear mean model (mm).

GA (wk)	t (days)	Pred. mean	Pred. SD	P5	P50	P95
18	129	9.76	1.13	7.91	9.76	11.62
19	136	10.89	1.36	8.66	10.89	13.12
20	143	12.02	1.58	9.42	12.02	14.63
21	150	13.15	1.81	10.17	13.15	16.13
22	157	14.28	2.04	10.93	14.28	17.63
23	164	15.41	2.26	11.68	15.41	19.13
24	171	16.54	2.49	12.44	16.54	20.64
25	178	17.67	2.72	13.19	17.67	22.14

Centiles calculated as $\mu(t) \pm 1.645 \cdot \sigma(t)$; t = gestational age in days at the midpoint of each week. GA, gestational age; Pred., predicted; SD, standard deviation.

Table S3. Bootstrap confidence intervals for model coefficients and the fitted median curve

Percentile-based 95% confidence intervals from 1000 bootstrap resamples drawn with replacement at the pregnancy level. Both the cubic mean model and the gestational-age-specific variance model were refitted in every resample; model selection was not repeated. t denotes gestational age in days.

Table S3a. Cubic mean model, μ(t).

Coefficient	Estimate	95% CI
Intercept	−155.712390	−333.834480 to 8.955537
t	3.104289	−0.137570 to 6.665690
t^2	−0.01985762	−0.043287 to 0.001274
t^3	0.0000444422	−0.0000030 to 0.0000957

Confidence intervals for the individual polynomial terms are wide and cross zero, reflecting collinearity among the powers of t; this does not indicate instability of the fitted curve (see Table S3c).

Table S3b. Variance model, before √(π/2) scaling.

Coefficient	Estimate	95% CI
Intercept	−2.351178	−3.183274 to −1.503333
t	0.025294	0.019581 to 0.031019

The slope coefficient excluded zero, supporting gestational-age-specific variance modelling.

Table S3c. Bootstrap 95% confidence interval for the fitted median curve.

GA (wk)	t (days)	Predicted median	95% CI
18	129	9.69	9.25 to 10.09
19	136	10.98	10.81 to 11.14
20	143	12.09	11.99 to 12.20
21	150	13.13	13.04 to 13.21
22	157	14.18	14.05 to 14.31
23	164	15.33	15.14 to 15.51
24	171	16.68	16.35 to 17.01
25	178	18.33	17.59 to 19.12

GA, gestational age; CI, confidence interval.

All analyses were performed in Python 3.12 using the NumPy, SciPy, and statsmodels libraries.
