# Supplementary material for: Gestational Age-Specific Reference Ranges and Internally Validated Z-Score Equations for Fetal Thymus Transverse Diameter at 18 + 0–25 + 6 Weeks of Gestation
Source: Diagnostics (Basel). 2026 Sep 14;16(18):2973. doi: 10.3390/diagnostics16182973 (PMC13605929; doi:10.3390/diagnostics16182973)
Supplement: Supplementary file 1 [file diagnostics-16-02973-s001.zip › 06_Supplementary_S4-S5.pdf]

## Supplementary Tables S4–S5

Gestational Age–Specific Reference Ranges and Internally Validated Z-Score Equations for Fetal Thymus Transverse Diameter at 18+0–25+6 Weeks of Gestation

**Table S4. Sensitivity of the model-derived reference values for fetal thymus transverse diameter (mm) to exclusion of individual maternal comorbidity categories and to their combined exclusion, 18+0–25+6 weeks. Each scenario was refitted independently using the same cubic mean model and gestational-age-specific variance model as the primary analysis.**

| Scenario | Parameter | n | 18 wk | 19 wk | 20 wk | 21 wk | 22 wk | 23 wk | 24 wk | 25 wk |
|---|---|---|---|---|---|---|---|---|---|---|
| Primary cohort | P50 (mm) | 2,103 | 9.69 | 10.98 | 12.09 | 13.13 | 14.18 | 15.33 | 16.68 | 18.33 |
| | Predicted SD (mm) | | 1.14 | 1.36 | 1.59 | 1.81 | 2.03 | 2.25 | 2.47 | 2.70 |
| | P5 (mm) | | 7.81 | 8.73 | 9.48 | 10.15 | 10.84 | 11.63 | 12.61 | 13.89 |
| | *ΔP5 vs primary (mm)* | | — | — | — | — | — | — | — | — |
| Excl. diabetes (type 1/2 and GDM) | P50 (mm) | 2,047 | 9.71 | 10.98 | 12.09 | 13.13 | 14.19 | 15.36 | 16.73 | 18.38 |
| | Predicted SD (mm) | | 1.15 | 1.37 | 1.59 | 1.81 | 2.03 | 2.25 | 2.47 | 2.69 |
| | P5 (mm) | | 7.82 | 8.73 | 9.48 | 10.16 | 10.85 | 11.66 | 12.66 | 13.95 |
| | *ΔP5 vs primary (mm)* | | *+0.01* | *-0.00* | *-0.00* | *+0.00* | *+0.02* | *+0.03* | *+0.05* | *+0.06* |
| Excl. hypertensive disorders (chronic HT and GHT) | P50 (mm) | 2,066 | 9.67 | 10.96 | 12.08 | 13.12 | 14.18 | 15.34 | 16.71 | 18.38 |
| | Predicted SD (mm) | | 1.14 | 1.36 | 1.58 | 1.80 | 2.02 | 2.25 | 2.47 | 2.69 |
| | P5 (mm) | | 7.80 | 8.73 | 9.48 | 10.16 | 10.85 | 11.65 | 12.65 | 13.95 |
| | *ΔP5 vs primary (mm)* | | *-0.01* | *-0.00* | *+0.00* | *+0.01* | *+0.01* | *+0.02* | *+0.03* | *+0.06* |
| Excl. thyroid disease | P50 (mm) | 1,858 | 9.67 | 10.99 | 12.11 | 13.13 | 14.17 | 15.33 | 16.73 | 18.48 |
| | Predicted SD (mm) | | 1.15 | 1.37 | 1.59 | 1.81 | 2.03 | 2.24 | 2.46 | 2.68 |
| | P5 (mm) | | 7.78 | 8.74 | 9.50 | 10.16 | 10.84 | 11.64 | 12.68 | 14.07 |
| | *ΔP5 vs primary (mm)* | | *-0.03* | *+0.01* | *+0.01* | *+0.01* | *-0.00* | *+0.01* | *+0.07* | *+0.18* |
| Excl. IVF conception | P50 (mm) | 2,048 | 9.64 | 10.96 | 12.09 | 13.13 | 14.17 | 15.32 | 16.66 | 18.29 |
| | Predicted SD (mm) | | 1.14 | 1.36 | 1.59 | 1.81 | 2.04 | 2.26 | 2.49 | 2.71 |
| | P5 (mm) | | 7.77 | 8.72 | 9.48 | 10.15 | 10.82 | 11.60 | 12.57 | 13.83 |
| | *ΔP5 vs primary (mm)* | | *-0.04* | *-0.02* | *-0.00* | *-0.01* | *-0.02* | *-0.03* | *-0.05* | *-0.06* |
| Excl. all four categories combined | P50 (mm) | 1,733 | 9.60 | 10.95 | 12.09 | 13.13 | 14.18 | 15.37 | 16.79 | 18.56 |
| | Predicted SD (mm) | | 1.15 | 1.37 | 1.59 | 1.81 | 2.02 | 2.24 | 2.46 | 2.68 |
| | P5 (mm) | | 7.71 | 8.70 | 9.48 | 10.16 | 10.86 | 11.68 | 12.74 | 14.16 |
| | *ΔP5 vs primary (mm)* | | *-0.11* | *-0.03* | *-0.00* | *+0.01* | *+0.02* | *+0.05* | *+0.13* | *+0.27* |

*P50, model-derived median; P5, model-derived 5th centile, calculated as $\mu(t) - 1.645\cdot\sigma(t)$ with $t$ = gestational age in days at the midpoint of each week, identical to the method used for Table 4 of the main text. The primary-cohort rows reproduce Table 4 exactly. Exclusions are applied to all pregnancies carrying the condition, and each scenario is independent rather than cumulative. No single-condition exclusion shifted P5 by more than 0.18 mm; combined exclusion of all four categories shifted P5 by at most 0.27 mm. The maximum shift occurred at 25 weeks in every scenario. Predicted values are shown rounded to two decimal places, whereas all differences quoted in the main text were calculated from unrounded model predictions; differences derived from the rounded values shown may therefore differ by up to 0.01 mm.*

**Table S5. Gestational-age-adjusted fetal thymus Z-score by mutually exclusive maternal comorbidity category, compared with the comorbidity-free reference group.**

| Group | n | Mean Z | SD | Mean difference vs. reference | 95% CI | p | p (Holm) | GA-trend slope | GA-trend p |
|---|---|---|---|---|---|---|---|---|---|
| Comorbidity-free (reference) | 1,733 | +0.003 | 1.001 | — | — | — | — | +0.0109 | 0.550 |
| Diabetes only (type 1/2 and gestational) | 47 | -0.099 | 1.057 | -0.101 | -0.415 to +0.212 | 0.519 | 1.000 | -0.0819 | 0.380 |
| Hypertensive disorders only (chronic and gestational) | 24 | +0.150 | 1.119 | +0.148 | -0.327 to +0.623 | 0.526 | 1.000 | -0.3077 | 0.061 |
| Thyroid disease only | 232 | -0.034 | 1.033 | -0.037 | -0.178 to +0.105 | 0.610 | 1.000 | -0.0161 | 0.786 |
| IVF conception only | 44 | +0.166 | 0.960 | +0.164 | -0.132 to +0.459 | 0.271 | 1.000 | -0.0775 | 0.518 |
| Multiple comorbidities | 23 | -0.118 | 0.981 | -0.121 | -0.547 to +0.306 | 0.563 | 1.000 | +0.0700 | 0.659 |

*Z-scores were derived from the final reference equations of the main analysis. Groups are mutually exclusive: each pregnancy contributes to one category only, and the six categories sum to the full analytical cohort (n = 2,103). Mean differences were calculated against the comorbidity-free reference group by Welch's two-sample t-test, with 95% confidence intervals based on the Welch–Satterthwaite degrees of freedom; differences were computed from unrounded group means and may therefore differ by up to 0.001 Z from the difference of the rounded means shown. p (Holm), p-value after Holm step-down correction across the five comparisons. GA-trend, simple linear regression of Z-score on gestational age in weeks within each category; slope expressed in Z per week. Global comparison across all six categories: Welch ANOVA F = 0.536 (df 5, 87.3), p = 0.748; Kruskal–Wallis H = 2.739 (df 5), p = 0.740.*
